# The Deployment of Carbon Monoxide Wireless Sensor Network (CO-WSN) for Ambient Air Monitoring

**DOI:** 10.3390/ijerph110606246

**Published:** 2014-06-16

**Authors:** Chaichana Chaiwatpongsakorn, Mingming Lu, Tim C. Keener, Soon-Jai Khang

**Affiliations:** Department of Biomedical, Chemical, and Environmental Engineering, University of Cincinnati, 2901 Woodside Dr., Cincinnati, OH 45221, USA; E-Mails: chaichana@gmail.com (C.C.); mingming.lu@uc.edu (M.L.); soon-jai.khang@uc.edu (S.-J.K.)

**Keywords:** carbon monoxide, wireless sensor network, monitoring, traffic, intersection

## Abstract

Wireless sensor networks are becoming increasingly important as an alternative solution for environment monitoring because they can reduce cost and complexity. Also, they can improve reliability and data availability in places where traditional monitoring methods are difficult to site. In this study, a carbon monoxide wireless sensor network (CO-WSN) was developed to measure carbon monoxide concentrations at a major traffic intersection near the University of Cincinnati main campus. The system has been deployed over two weeks during Fall 2010, and Summer 2011–2012, traffic data was also recorded by using a manual traffic counter and a video camcorder to characterize vehicles at the intersection 24 h, particularly, during the morning and evening peak hour periods. According to the field test results, the 1 hr-average CO concentrations were found to range from 0.1–1.0 ppm which is lower than the National Ambient Air Quality Standards (NAAQS) 35 ppm on a one-hour averaging period. During rush hour periods, the traffic volume at the intersection varied from 2,067 to 3,076 vehicles per hour with 97% being passenger vehicles. Furthermore, the traffic volume based on a 1-h average showed good correlation (R^2^ = 0.87) with the 1-h average CO-WSN concentrations for morning and evening peak time periods whereas CO-WSN results provided a moderate correlation (R^2^ = 0.42) with 24 hours traffic volume due to fluctuated changes of meteorological conditions. It is concluded that the performance and the reliability of wireless ambient air monitoring networks can be used as an alternative method for real time air monitoring.

## 1. Introduction

Carbon monoxide (CO) is an odorless, tasteless and colorless gas that can be found at dangerous concentrations both indoors and outdoors [1]. Ambient CO comes primarily from automobile exhaust and high concentrations have been reported in enclosed garages, along roadways, and near intersections. In 2011, U.S. EPA reported that on-road and non-road vehicles contribute approximately 41 million short tons which accounts for 50% of the national carbon monoxide emissions [2]. CO can directly affect the health of people who work or live nearby these areas. After inhalation into the respiratory system, it eventually prohibits hemoglobin (Hb) in blood cells binding and carrying oxygen molecules because it reacts with hemoglobin faster than oxygen does [3]. Inhaling at high concentration of CO can result in dizziness, headaches, unconsciousness, and even death [4]. Therefore U.S. EPA has established the National Ambient Air Quality Standards (NAAQS) for CO in 1971. Then, U.S. EPA decided to retain the current standard at 9 ppm for 8-h average and 35 ppm for 1-h average after the most recent review of NAAQS for CO in 2011 [5]. 

Recently many studies have been accomplished to scrutinize the correlation between CO exposure and traffic. Kaur *et al.* [6] have studied the personal exposure and the carbon monoxide at the street canyon intersections. The results indicated that the personal CO exposure levels were high during morning rush hours. Chan *et al.* [7] have indicated that the CO concentrations from vehicular exhausts contributed more CO levels to nearby pedestrians than to people who are in vehicles. As a result of the studies above, and an increasing community concern to CO exposure due to the implementation of more bicycle lanes, more pedestrians and more mixed traffic as a consequence of the driving-less initiatives, it is necessary to have enough 24-h CO monitoring stations covering potential hot-spot areas. 

In order to measure CO concentrations continuously, U.S. EPA recommends non-dispersive infrared (NDIR) as a standard reference method. The principle of this technique is based on absorption of infrared radiation by CO molecules in the wavelength of 4.7 µm region [8]. This method has several advantages. For example, its measurement is generally not affected by an ambient temperature, and it provides a good sensitivity to the broad concentration range with a short response time. However, this method requires a substantial infrastructure including calibration gases, pumps, monitoring stations and other peripheral equipment along with a rather expensive analyzer [9]. Because of the high expense, these measurements can only be conducted at very limited sites. For instance, in the United States, there were approximately 328 CO monitoring stations as of May 2011 but only 52 CO monitoring sites are operating near roadways [5]. In addition, many previous studies have shown that the pollutant measurement from fixed monitoring stations provide underestimated measurements compared with the actual exposure levels [10,11]. This error was caused by the variation in sampling heights, the distance from the hot spot area, and the insufficient sampling rate.

Recent advances in wireless sensor networks (WSNs) have shown an alternative solution for monitoring ambient air quality. Some WSN systems have been developed for short-term and long-term operations. For instance, Chung and Oh [12] developed a wireless sensor to monitor the indoor CO_2_ concentrations. A significant advancement of this study lies in that the circuit module has various enlargements for installing other sensors such as humidity, CO_2_, and flying dust sensors, *etc.* Furthermore, Cordova-Lopez *et al.* [13] have integrated a Geography Information System (GIS) with a wireless sensor network using CO, CO_2_, NO, NO_2_, SO_2_, and hydrocarbon sensors for monitoring tailpipe emissions from vehicle exhaust. They found that, during idle conditions when the car is waiting for a red traffic light, hydrocarbons and CO_2_ were the lowest compared with other pollutants while at a speed of 70 km/h nitrogen oxides (NO_x_) levels reached the highest concentration 100 ppm,. However, no ambient evaluation has been performed for monitoring carbon monoxide with WSNs. 

In this study, a CO-WSN system is comprised of a carbon monoxide sensor, a humidity sensor, and a temperature sensor integrated with a data acquisition board, a wireless communication system and a solar panel for monitoring CO concentrations continuously without charging. According to the preliminary experimental results, the lowest detection limit of the CO sensor is 0.1 ppm which is equivalent to the U.S. EPA reference method. Sensor will provide the advantage of flexibility in a deployment, a lower operation and a maintenance cost compared with the NDIR reference method, and will offer much more real time information about the spatial and temporal variations of ambient CO concentrations. This type of information is crucial for use by the transportation planners, environmental regulators, and the general public.

## 2. Materials & Methods

### 2.1. Components of CO-WSN

**Figure 1:**
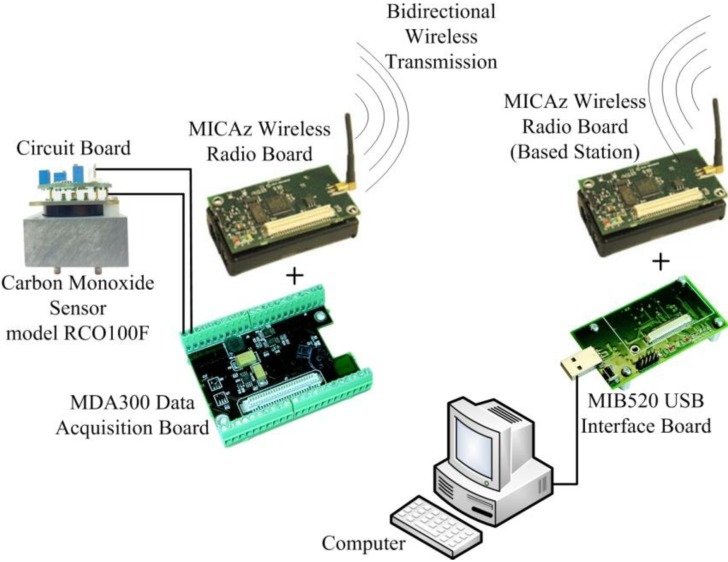
Components of the CO wireless sensor network (CO-WSN).

A CO-WSN is comprised of five functional units: an analog sensor unit, a data acquisition board, a wireless radio module, a solar panel unit, and a radio antenna (Figure 1).

#### 2.1.1. Analog Sensor Unit

Each analog sensor unit consists of a CO sensor and a signal amplifying board. There are three different options for the CO sensor: chemo-optical, semiconductor and electro-chemical. Among them, the electro-chemical sensors are typically found to report more accurate CO concentrations and are inexpensive in comparison with the others. Electro-chemical sensors generally use an acid as electrolyte and platinum (Pt) as a catalyst to react with the carbon monoxide gas and give off electrons. The principle chemical reactions inside the sensor are shown as follow [14,15,16]:

At working electrode: CO + H_2_O **→** CO_2_ + 2H^+^ + 2e^−^ (1)

At counter electrode: ½O_2_ + 2H^+^ + 2e^−^**→** H_2_O (2)

Overall reaction: CO + ½O_2 _**→** CO_2_ (3)

At the working electrode, carbon monoxide reacts with water and provides electrons in proportion to the CO concentration while oxygen reacts with hydrogen ions and electrons and gives off water as a product at the counter electrode. The current generated at the working electrode, usually reported as voltages, can be used to determine CO concentration in the ambient. In our system design, the CO sensor connected to the data acquisition board (MDA 300CA) that can convert an analog signal of the CO sensor to a digital signal and then, transmitted to the MicaZ base station (MIB520) by a MicaZ radio transceiver module. Furthermore, the temperature and relative humidity (RH) can be detected by sensors that are located on the data acquisition board (MDA 300CA). In this study a three electrode electrochemical CO sensor model RCO100F (KWJ Engineering Inc., Newark, CA, USA) was customized and assembled into the wireless network system. The sensor specification is also shown in Table 1.

**Table 1:** CO sensor model RCO100F specifications.

Characteristics	Specifications
Size (W × L × H)	3.2 × 3.2 × 2.0 cm
Measuring Range	0–100 ppm
Measuring Principle	Electrochemical Oxidation of CO
Onboard Filter	To remove SO_x_, NO_x_ & H_2_S
Output Signal, Zero, 25 °C	<±1 ppm equivalent maximum
Output Signal, Span, 25 °C	0.20 ± 0.03 mA/ppm
Lower Detection Limit	<0.5 ppm (depends on circuitry)
Resolution	°.5 ppm (depends on circuitry)
Repeatability	1% of Signal
Output Linearity	Linear
Response Time (t-90)	<30 s typical at 20 °C
Long Term Drift—Span	Output Signal ≤ ± 2% of reading per month
Operating Temperature Range	−20 to 50 °C (0–35 °C recommended)

#### 2.1.2. Data Acquisition Board

In order to measure the three signals, CO concentration, temperature and humidity simultaneously, we used MDA300CA, an environmental data acquisition module that attaches directly to the mote and has the capability to interface with external sensors. Additionally, this 12 bit data acquisition board has a ADC sampling time 782 μs which is compatible with both analog and digital inputs with a high precision analog to digital converter [17]. 

#### 2.1.3. Wireless Radio Module

The wireless radio modules usually include a microcontroller, a flash memory, a wireless transceiver and interface to external sensors. The wireless radio modules are also known as “motes”. These motes are AA battery-powered wireless devices whose primary function is to send and receive data. They have a built-in processor that is capable of running TinyOS, an open source operating system designed for embedded wireless networks. At present there are many prototype and commercial wireless radio modules available. Some products integrate specific sensors on the wireless radio modules to make it a self-contained wireless sensor node. Other sensors provide only the interface for the user to connect their customized sensors. In this study, MicaZ self-contained the wireless radio module (Crossbow Inc., Milpitas, CA, USA) is chosen to transmit and receive the sensor data by Mica radio and gateway node respectively. The wireless radio results can be viewed on a computer by using a Moteview program version 2.0F.

#### 2.1.4. Solar Panel Unit and Radio Antenna

**Figure 2:**
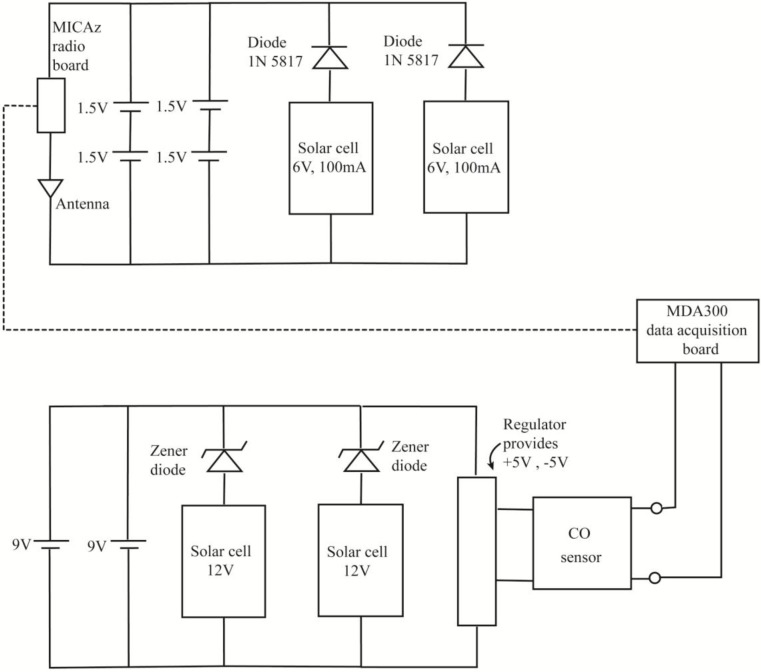
A CO wireless sensor circuit diagram.

To receive continuous signals from CO sensors in a long-term operation without battery change, solar panels have been considered as an additional important component assembled with the CO-WSN. A CO wireless sensor circuit diagram is shown in Figure 2. Each unit of CO wireless sensor consists of a MicaZ Radio Transceiver Module, Antenna, AA rechargeable batteries, “9V” rechargeable batteries, 2 sets of 6 V, 100 mA thin film solar panels, 2 sets of 12 V, 100 mA thin film solar panels, a three electrode electrochemical CO sensor model RCO100F, and a MDA 300 data acquisition board (Figure 2).

For the radio antenna module, three different varieties of antennas and wireless radios were utilized, Mica2 433 MHz, Micaz, and MicaZ. Each was tested to determine the optimum distance in order to maximize a received data efficiency. The experimental results indicated that MicaZ with the external 9.0 dbi antenna had the maximum transmission capability at a distance of more than 300 m. Prior to the utilization of the CO-WSN for data collection, the wireless sensors were calibrated against the NDIR standard method, and a preliminary deployment was implemented at the university main campus for data collection and analysis

### 2.2. CO Wireless Sensor Calibration Method

**Figure 3:**
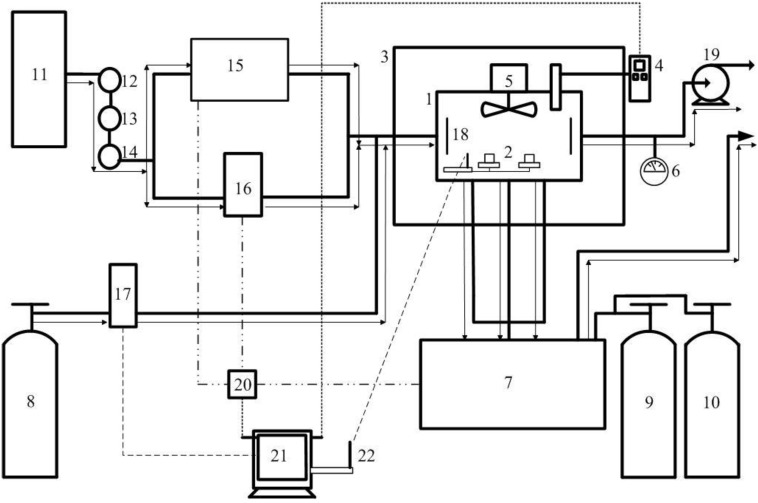
Schematic diagram of CO calibration system.

Notes: 1. Exposure chamber; 2. CO wireless sensor; 3. Incubator; 4. Thermo-hygrometer; 5. Mixer; 6. Pressure sensor; 7. CO-NDIR analyzer; 8. 100% CO; 9. Standard CO 40 ppm; 10. Standard CO 10 ppm; 11. Air pump; 12. Molecular sieve; 13. Activated charcoal; 14. Hopcalite /Ascarite; 15. Flow temperature humidity control system; 16. Mass flow controller No.1; 17. Mass flow controller No.2; 18. Baffle; 19. Pump; 20. Data acquisition system; 21. Computer; 22. Wireless sensor receiver board; → Flow direction.

Each assembled CO wireless sensor unit requires a calibration and a validation processes in the laboratory prior to use for continuous monitoring. An environmental exposure chamber (Figure 3) was constructed for testing the sensor under different CO concentration, temperature, and humidity conditions. Temperature and humidity inside the chamber were measured by a NIST traceable thermo-hygrometer (TSI Inc. model 8722; Shoreview, MN, USA), and CO concentration was determined by the NDIR analyzer (Dasibi model 3030; Glendale, CA, USA). Carbon monoxide certified standard gas at concentration of 10 and 40 ppm were used to enumerate concentration levels of CO for measurements by the NDIR and CO wireless sensor. 

The environmental exposure chamber size (W × L × H): 0.25 × 0.45 × 0.25 m uses inert acrylic polymer which does not react with the CO or other hazardous air pollutants. It was designed to create a homogenous distribution of CO concentration with different levels of temperature and relative humidity in the range of 5–40 °C and 0%–80% RH. In order to formulate the precise CO concentration, the purified air was provided by using an air generator in the laboratory. The air flow was passed through a molecular sieve containing hopcalite (a mixture of copper and manganese oxides), ascarite (non-fibrous silicate carrier coated with sodium hydroxide), and activated carbon columns in order to remove moisture, carbon monoxide, carbon dioxide and residue hydrocarbons respectively. The desired CO concentration was then produced by diluting pure CO with purified air to the designated value. The humidity in the chamber was varied by a flow-temperature-humidity control system (MNR model HCS-401; Livermore, CA, USA). The chamber was placed in an incubator (Jordon refrigerator model FT1W-TRGBOD; Philadephia, PA, USA) in order to maintain constant temperature in the range of 0–40 °C. 

**Figure 4:**
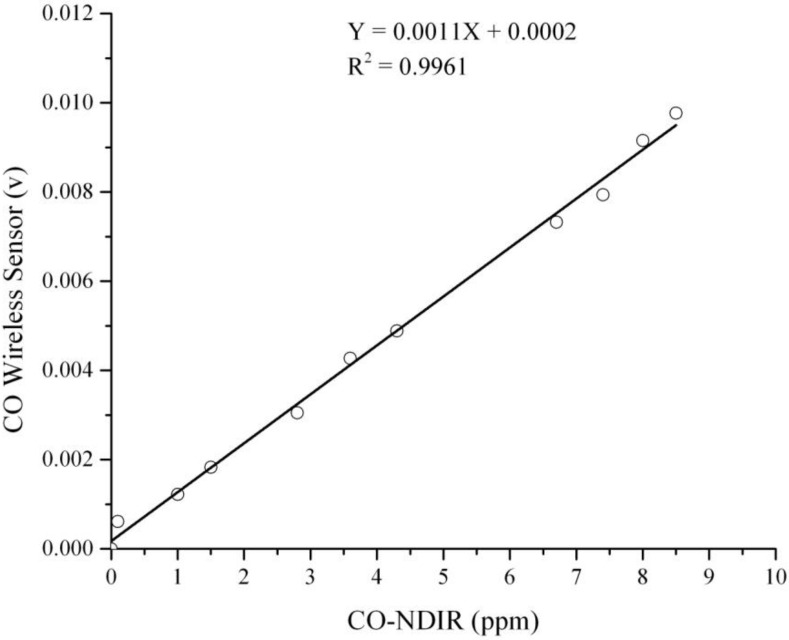
Calibration curve of CO wireless sensor at 25 °C and 50% RH.

**Figure 5:**
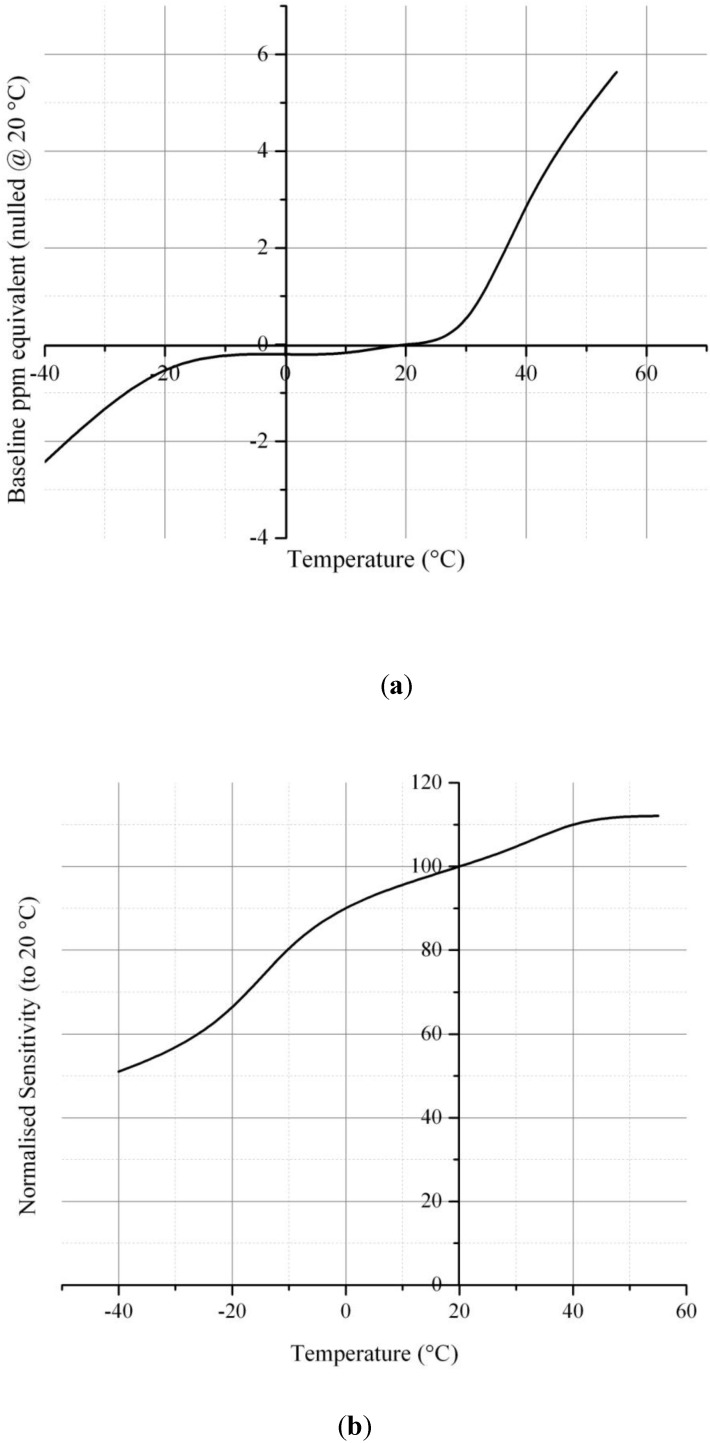
Temperature gradient effect on CO sensor RCO100F. (**a**) Sensor output *vs*. temperature relative to 20 ºC; (**b**) Sensor sensitivity relative to 20 ºC.

Two sets of experimental results from the data acquisition system were recorded and transferred to a computer for converting analog signals to digital outputs with a sampling rate of 15 s/data point. The first set of results is the analog signals from the data acquisition system obtained from the CO, temperature and humidity sensors. The second set of results is the reference data acquired from the NDIR analyzer and NIST traceable thermo-hygrometer. The linear regression equations were developed from the correlation between CO wireless sensor output and CO-NDIR to get the actual CO concentration. The example calibration curve of CO sensor at the specific temperature and humidity condition that used for this study is shown in Figure 4. In addition, Figure 5 presents the temperature gradient effect on CO sensor RCO100F was performed by Vinay Patel (KWJ Engineering Inc., Newark, CA, USA).

In addition, it is important to note that the adjusted CO sensor output at 0.6 mV is corresponding to the lowest voltage level of the MDA300CA which equals to the output from the CO sensor at carbon monoxide concentration of 0.1 ppm. The voltage of CO sensor after connected with an analog-to-digital converter (ADC) can be converted by using the following equation [18]:

Voltage = 2.5 × ADC reading/4096
(4)

### 2.3. The Deployment of CO Wireless Sensor

**Figure 6:**
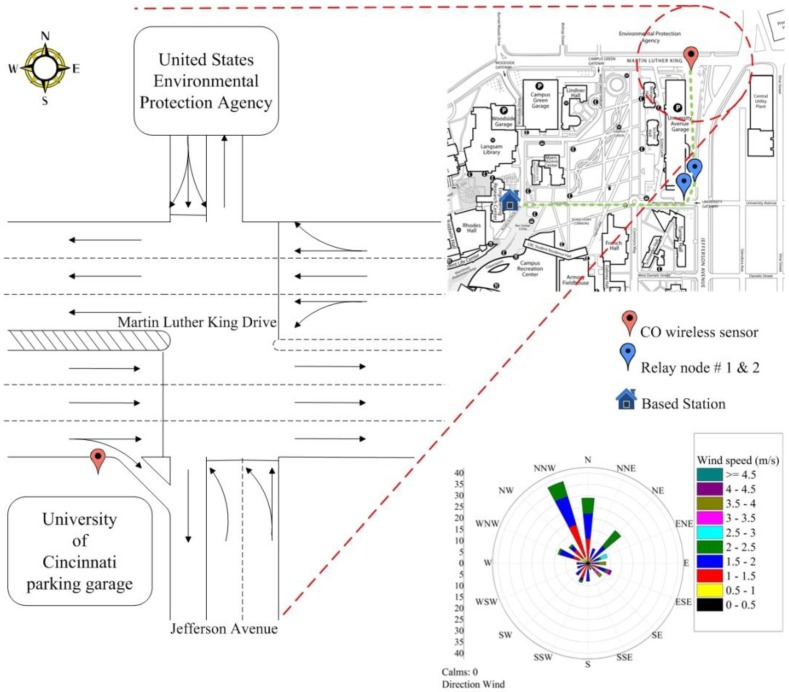
Jefferson-Martin Luther King Intersection.

In this study, two relay nodes and one CO wireless sensor unit were installed and deployed at a height of 2.1 m above the ground on the southwest of the Jefferson Avenue—Martin Luther King Drive Intersection (at an elevation of 239.3 m) (Figure 6). The CO wireless sensor unit was located at 0.5 m from a curb side of the Martin Luther King Drive. The distance of the relay nodes #1 and #2 from the based station is approximately 365.8 m while the CO wireless sensor box is located at about 470.9 m far from the based station. Each unit recorded the data every 15 s and transmitted the data 24 h continuously to the base station located at 4th floor of the Engineering Research Center (ERC) building. 

### 2.4. Meteorological Data

Two meteorological towers are used as a reference in this study. The first one is located at the Hamilton County Department of Environmental Services (HCDES) which is approximately 933 m far from the intersection (at an elevation of 259 m). The sampling rate of this tower is 1 h. The second tower is located at the Rhodes Hall Building, University of Cincinnati (at an elevation of 270.4 m). It is comprised of a wind sensor model 034B, and a temperature/humidity sensor model 083D (The Met One Instruments Inc., Grants Pass, OR, USA). Wind speed and wind direction, temperature, and relative humidity have been recorded automatically to a data logger at a sampling rate of 1 min. The example of windrose plot from 16–23 September 2010 is shown in Figure 6.

### 2.5. CO & Traffic Data Collection and Analysis

CO and traffic data were collected simultaneously 24 h a day and focused on the morning (7–9 a.m.) and evening (4–6 p.m.) peak traffic hours prior and after the school started. The Jefferson Avenue—Martin Luther King Drive Intersection is a major intersection located at the northwest corner of the University of Cincinnati west campus. 

The total numbers of vehicles at this intersection were recorded by each approach and were labeled as northbound (NB), southbound (SB), eastbound (EB), and westbound (WB). The data were recorded on a one minute basis by using a handheld traffic data collector (Jamar Technologies, Inc., model TDC-12; Hatfield, PA, USA). Traffic at the intersection was also recorded using a camcorder for record keeping and future data analysis. Traffic data was collected from 16–23 September 2010 during the morning (7–9 a.m.) and evening (4–6 p.m.) peak times. The traffic volume for each approach was categorized into two classes: passenger vehicle (PV) and heavy duty vehicle (HDV).

Figure 6 also represents the traffic pattern of the intersection. The WB and NB routes go toward the west campus of UC which is the main campus. The SB traffic directly comes from the U.S. EPA, whereas most of the EB traffic comes from I-75 and goes to the UC East campus (the medical/research campus) and other locations along the Martin Luther King Drive. 

## 3. Results and Discussion

CO wireless sensor and two relay boxes were installed for two weeks in order to obtain real time 24-h CO concentrations as well as temperature, and relative humidity data at the Jefferson—Martin Luther King intersection. 

**Figure 7:**
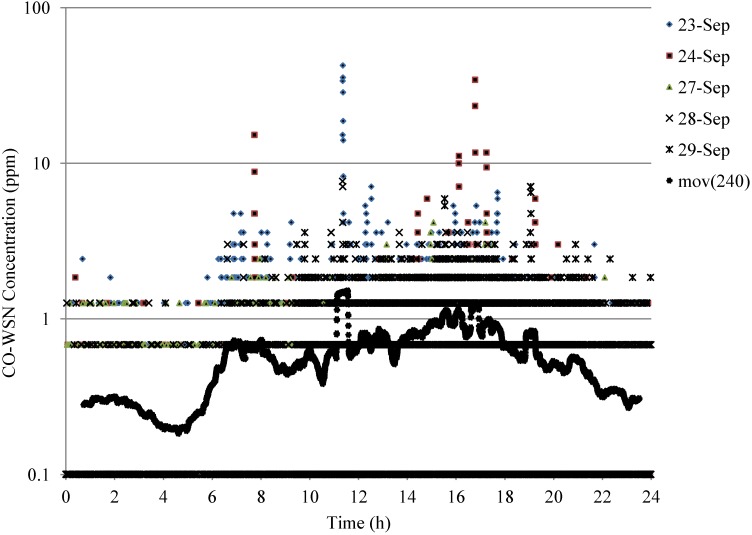
Example of 24-h CO concentrations at the Jefferson—Martin Luther King Intersection—weekdays with 240 points moving average.

**Figure 8:**
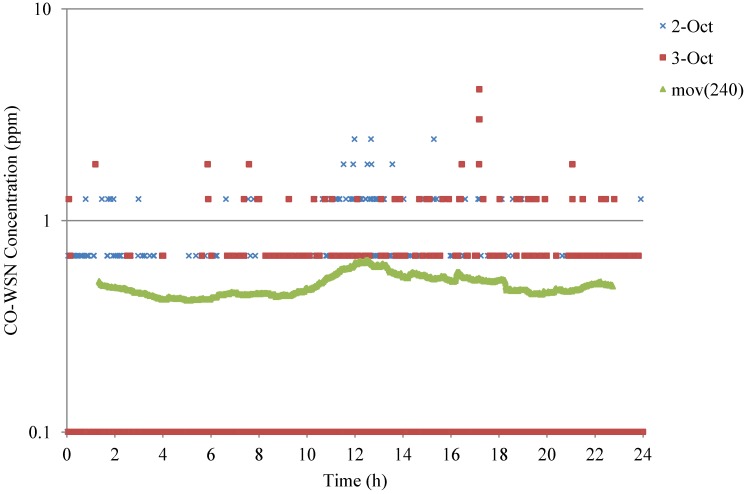
Example of 24-h CO concentrations at the Jefferson—Martin Luther King Intersection—weekend with 240 points moving average.

Continuous data was obtained every 15 s from the base station located at 4th floor of the Engineering Research Center building. Examples of 15 sec-CO data during weekdays for a 24 h period at the intersection are shown in Figure 7 and weekend data is shown is Figure 8. 

Figure 7 and Figure 8 show the weekdays and weekend trends respectively of the 24 h CO concentrations and the moving average of 240 points is approximately an one-hour average. The a.m. and p.m. peaks can be clearly observed on weekdays, which are likely corresponding to the office hours of the west campus and due to the commute trips of UC faculty, staff and students, whereas there is no peak found during the weekend. High concentrations were also recorded around noon time, which can be associated with lunch hour breaks. The p.m. peak is slightly higher than the a.m. peak. The maximum 5 min average CO concentration (10.6 ppm) was observed on 23 September 2010 around noon. The CO and traffic characterization will be further investigated for this period.

As shown in Table 2, the total number of vehicles during the morning and evening peak times ranged from 2,067–3,076 vehicles per hour. The majority of vehicles are passenger vehicles including motorcycles, SUVs, sedans, mini vans *etc.* whereas less than 3% is classified as heavy duty vehicles such as buses and trucks. During the first week of the fall quarter (week of 22 September), there was a 20% traffic increase from northbound and westbound during a.m. peak hour, which is likely due to many University of Cincinnati students returning to school at the beginning of new academic year.

**Figure 9:**
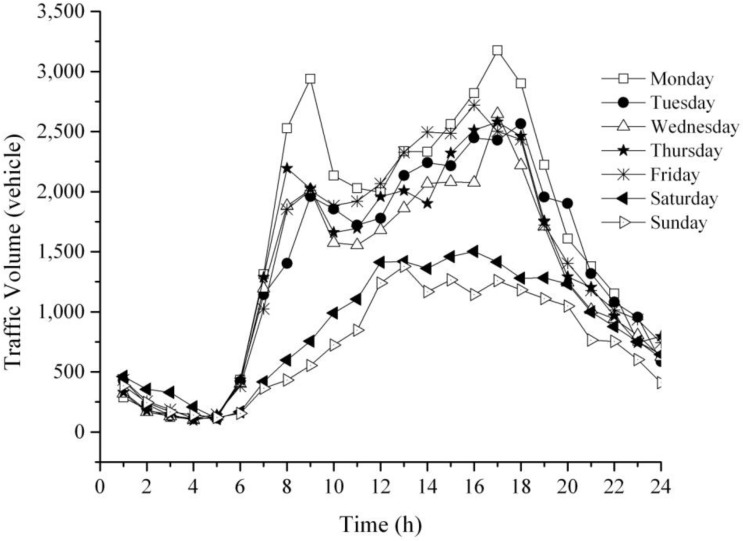
24-h traffic volume at the Jefferson—MLK Intersection.

Furthermore, 24-h traffic volume at the Jefferson—Martin Luther King intersection has been observed from Monday to Sunday during the summer (June–August) of 2011 and 2012 to investigate a traffic pattern. As illustrated in Figure 9, there are more than 30,000 vehicles passed through this intersection during weekdays and 15,000 vehicles during weekend. Two dominant peaks have been shown in morning and evening peak times of weekdays (Monday to Friday). These two peaks are analogous to a 24 h CO trend which is shown in Figure 7. Whereas, traffic volume during weekend (Saturday–Sunday) is only approximately 50% compared with weekdays period. Therefore, traffic volume peaks of weekend are flatter than regular weekdays which are corresponding to a 24-h CO pattern as shown in Figure 8.

**Table 2:** Vehicle characteristics at the Jefferson—Martin Luther King intersection.

Date and Time	Northbound (NB)		Southbound (SB)		Westbound (WB)		Eastbound (EB)		Traffic Volume (vph)
	PV (%)	HDV (%)	PV (%)	HDV (%)	PV (%)	HDV (%)	PV (%)	HDV (%)	
Monday 7–8 a.m.	176	3	8	1	607	37	1,320	38	
20 September 2010	(8.04)	(0.14)	(0.37)	(0.05)	(27.72)	(1.69)	(60.27)	(1.74)	2,190
Monday 8–9 a.m.	214	7	7	1	675	50	1,255	43	
20 September 2010	(9.50)	(0.31)	(0.31)	(0.04)	(29.97)	(2.22)	(55.73)	(1.91)	2,252
Tuesday 7–8 a.m.	192	5	9	0	657	26	1,330	40	
21 September 2010	(8.50)	(0.22)	(0.40)	(0.00)	(29.08)	(1.15)	(58.88)	(1.77)	2,067
Tuesday 8–9 a.m.	258	7	7	0	722	46	1,285	47	
21 September 2010	(10.88)	(0.30)	(0.30)	(0.00)	(30.44)	(1.94)	(54.17)	(1.98)	2,114
Wednesday 7–8 a.m.	292	4	4	0	756	30	1,274	37	
22 September 2010	(12.18)	(0.17)	(0.17)	(0.00)	(31.54)	(1.25)	(53.15)	(1.54)	2,397
Wednesday 8–9 a.m.	341	10	5	0	800	42	1,183	51	
22 September 2010	(14.02)	(0.41)	(0.21)	(0.00)	(32.89)	(1.73)	(48.64)	(2.10)	2,432
Thursday 7–8 a.m.	266	3	7	1(0.04)	705	30	1356	35	
23 September 2010	(11.07)	(0.12)	(0.29)		(29.34)	(1.25)	(56.43)	(1.46)	2,403
Thursday 8–9 a.m.	313	9	11	2	739	47	1,252	55	
23 September 2010	(12.89)	(0.37)	(0.45)	(0.08)	(30.44)	(1.94)	(51.57)	(2.27)	2,428
Thursday 4–5 p.m.	439 (14.27)	3	84	1	1,294	16	1,223	16	
16 September 2010		(0.10)	(2.73)	(0.03)	(42.07)	(0.52)	(39.76)	(0.52)	3,076
Thursday 5–6 p.m.	391	0	57	1	1,116	18	1,071	20	
16 September 2010	(14.62)	(0.00)	(2.13)	(0.04)	(41.74)	(0.67)	(40.05)	(0.75)	2,674
Monday 4–5 p.m.	396	0	83	0	1,179	0	1,044	0	
20 September 2010	(14.66)	(0.00)	(3.07)	(0.00)	(43.63)	(0.00)	(38.64)	(0.00)	2,702
Monday 5–6 p.m.	423	0	64	0	1,160	0	1,087	0	
20 September 2010	(15.47)	(0.00)	(2.34)	(0.00)	(42.43)	(0.00)	(39.76)	(0.00)	2,734
Tuesday 4–5 p.m.	437	2	88	24	1,220	0	1,097	21	
21 September 2010	(15.13)	(0.07)	(3.05)	(0.83)	(42.23)	(0.00)	(37.97)	(0.73)	2,889
Tuesday 5–6 p.m.	416	1	59	18	1,175	2	1,022	23	
21 September 2010	(15.32)	(0.04)	(2.17)	(0.66)	(43.26)	(0.07)	(37.63)	(0.85)	2,716
Wednesday 4–5 p.m.	447	1	73	1	1,278	24	1,103	25	
23 September 2010	(15.14)	(0.03)	(2.47)	(0.03)	(43.29)	(0.81)	(37.36)	(0.85)	2,952
Wednesday 5–6 p.m.	472	1	71	0	1,127	15	1,137	21	
23 September 2010	(16.60)	(0.04)	(2.50)	(0.00)	(29.63)	(0.53)	(39.98)	(0.74)	2,844

**Figure 10:**
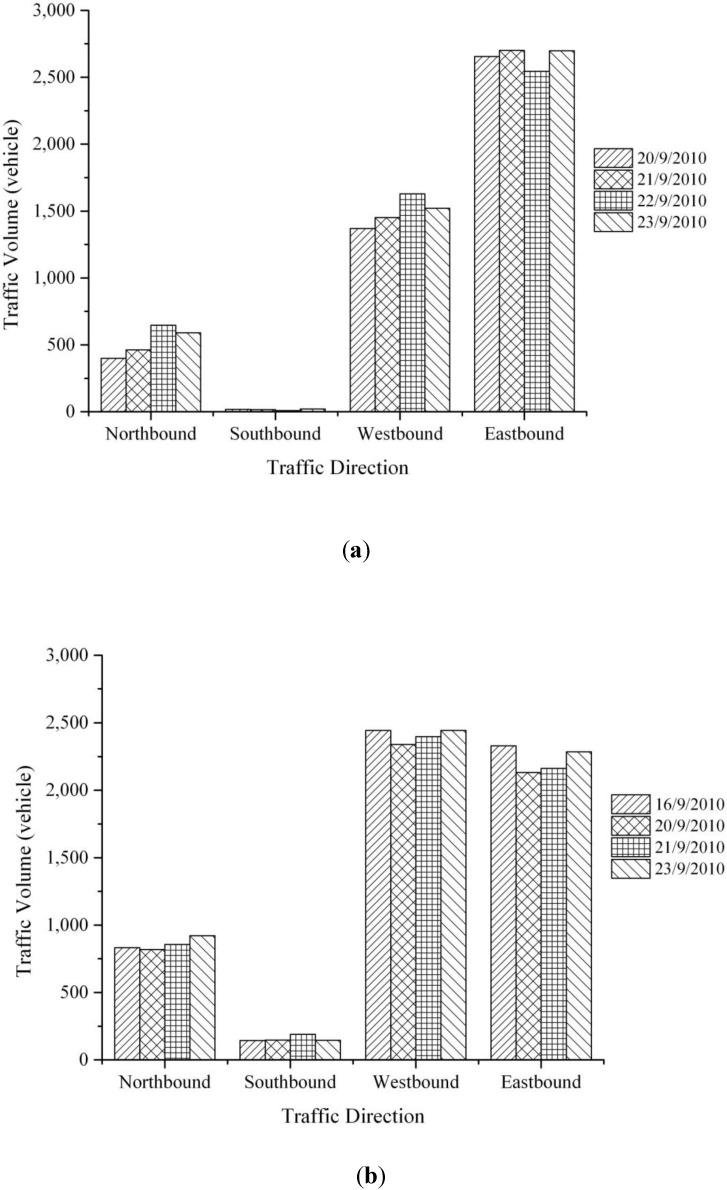
Traffic volume for each approach *vs.* a.m. & p.m. times. (**a**) 7–9 a.m.; (**b**) 4–6 p.m.

According to Figure 10a and b, a4–6 p.m. period presents higher traffic volumes than that of 7–9 a.m. period except for the eastbound direction. The eastbound and westbound approaches showed the highest number of vehicles passing this intersection, both along the Martin Luther King Drive. Martin Luther King Drive is well known as a major crosstown artery in Cincinnati area as it directly connects to the I-75, an interstate highway. Additionally, it links the west side of the city to the east, running through several historic uptown neighborhoods. The northbound and southbound sides had lower traffic volumes compared with the eastbound and the westbound sides even during peak time periods.

**Figure 11:**
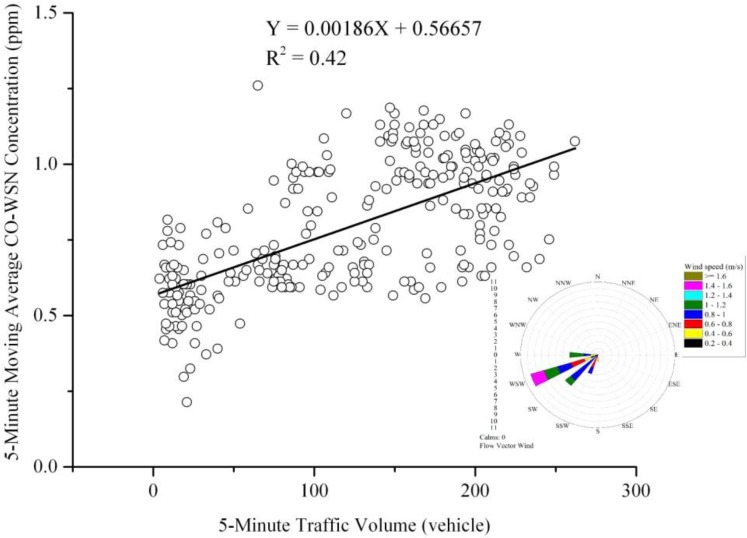
24-h CO-WSN concentrations.

Besides, Figure 11 represents a moderate correction (R^2^ = 0.42) between 5-min traffic volume and 5-min moving average CO-WSN concentration within 24 h. This could be explained by variations in meteorological conditions during the day, especially wind directions and wind speeds, the two factors known to affect the carbon monoxide dispersion.

**Figure 12:**
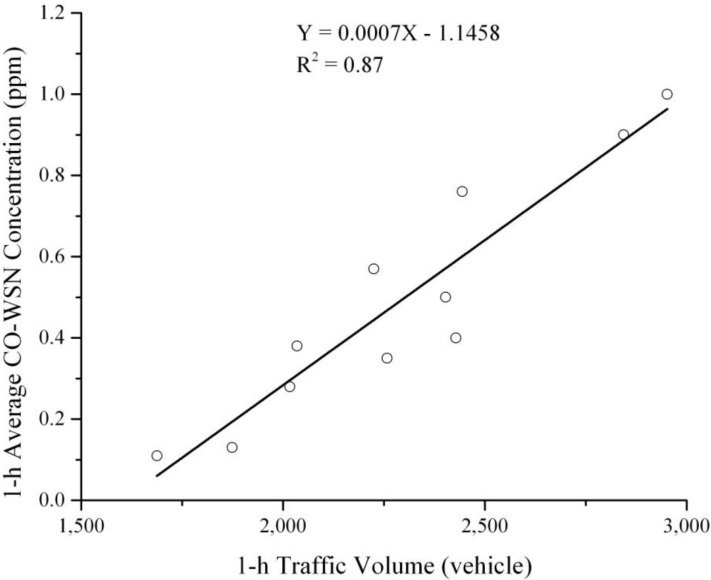
Relationship between 1-h traffic volume and 1-h average CO-WSN concentration during peak time periods.

As depicted in Figure 12, a high correlation between 1-h traffic volume and 1-h average CO-WSN concentration was observed during peak time periods (R^2^ = 0.87). This enforces the idea that on-road vehicles are major contributors to carbon monoxide concentration at the Jefferson—Martin Luther King intersection. 

**Figure 13:**
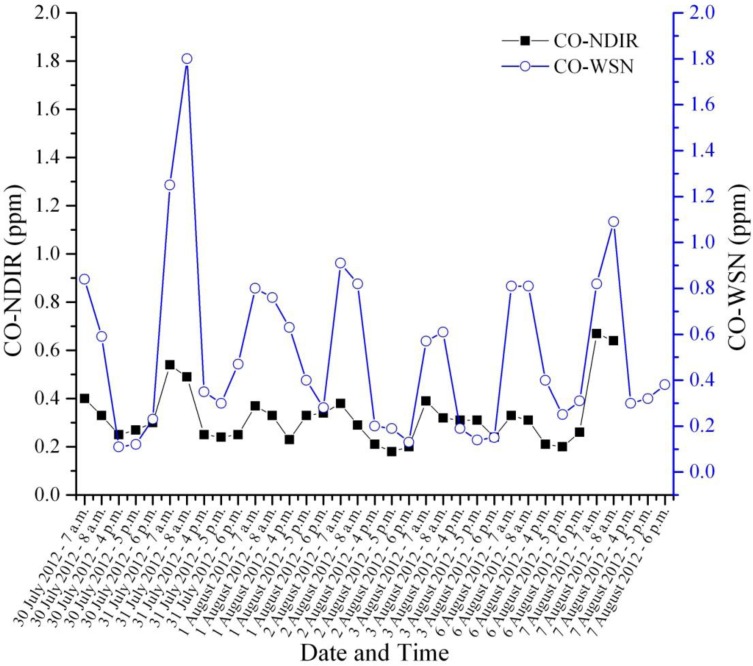
Comparison between CO-WSN and CO-NDIR results.

Furthermore, CO-WSN has been deployed at the Jefferson—Martin Luther King intersection and compared the results with that obtained from the CO-NDIR reference method from 30 July to 7 August 2012. This monitoring station is located at the Hamilton County Department of Environmental Services (HCDES) on the William Howard Taft Road. The distance between CO-WSN and the CO-NDIR is approximately 938 m. This is the only CO monitoring site in Cincinnati area. Figure 13 illustrates CO concentrations during weekdays morning (7–8 a.m.) and evening (4–6 p.m.) peak time periods. Apparently CO from the CO-WSN at the intersection provided higher concentrations than the CO-NDIR obtained from the HCDES monitoring station as a result of high traffic congestion.

## 4. Conclusions

In this study, the CO-WSN has developed and deployed at the Jefferson Avenue—Martin Luther King Drive intersection during Fall 2010 and Summer 2011–2012. The preliminary results show that The sensitivity of CO wireless sensor is comparable with the CO-NDIR reference method. The hourly average CO concentration also provides a good correlation with total vehicles that passed at the intersection during peak time periods. The CO-WSN can be used for ambient air monitoring in the long term operation by using the power from the sunlight, and also by connecting it with an internet server, it can provide a real time CO concentration level, temperature, and relative humidity to the local public.
